# Pelvic Organ Prolapse Quantification System (POP–Q) – a new era in pelvic prolapse staging

**Published:** 2011-02-25

**Authors:** C Persu, CR Chapple, V Cauni, S Gutue, P Geavlete

**Affiliations:** * Department of Urology, ‘Saint John’ Emergency Clinical Hospital, BucharestRomania; ** Department of Urology, Royal Hallamshire Hospital, Sheffield Teaching Hospital NHS Foundation Trust, SheffieldUK

**Keywords:** POP–Q, prolapse, staging, cystocele, rectocele

## Abstract

The prolapse of one or several pelvic organs is a condition that has been known by medicine since its early days, and different therapeutic approaches have been proposed and accepted. But one of the main problems concerning the prolapse of pelvic organs is the need for a universal, clear and reliable staging method.

Because the prolapse has been known and recognized as a disease for more than one hundred years, so are different systems proposed for its staging. But none has proved itself to respond to all the requirements of the medical community, so the vast majority were seen coming and going, failing to become the single most useful system for staging in pelvic organ prolapse (POP).

The latest addition to the group of staging systems is the POP–Q system, which is becoming increasingly popular with specialists all over the world, because, although is not very simple as a concept, it helps defining the features of a prolapse at a level of completeness not reached by any other system to date. In this vision, the POP–Q system may reach the importance and recognition of the TNM system use in oncology.

This paper briefly describes the POP–Q system, by comparison with other staging systems, analyzing its main features and the concept behind it.

## Introduction

Pelvic Organ Prolapse Quantification system (POP–Q) refers to an objective, site–specific system for describing, quantifying, and staging pelvic support in women [1]. It provides a standardized tool for documenting, comparing, and communicating clinical findings with proven interobserver and intraobserver reliability [2]. The POP–Q system gained the attention of the specialists all over the world, being approved by the International Continence Society (ICS), the American Urogynecologic Society (AUGS), and the Society of Gynecologic Surgeons for the description of female pelvic organ prolapse. It is the most common system used by gynecologists and urogynecologists, although other systems have been devised [3]. Nevertheless, its use is not yet accepted worldwide in routine care, while his ‘rival’, the Baden–Walker Halfway Scoring System is the next most commonly used system, as we'll see further in this article.

POP is a common and distressing condition. It occurs when there is a weakness in the supporting structures of the pelvic floor allowing the pelvic viscera to descend. While usually not life–threatening, prolapse is often associated with deterioration in quality of life and may contribute to bladder, bowel and sexual dysfunction. Extended life expectancy and an expanding elderly population mean that prolapse is an increasingly prevalent condition.

Symptoms associated with prolapse are often difficult to correlate with the anatomical site or severity of the ‘bulge’ and are often nonspecific [4]. Women with prolapse typically complain of the sensation of a ‘lump’ or vaginal ‘heaviness’, recurrent irritative bladder symptoms, voiding difficulty, incontinence or defecatory difficulty. Other symptoms such as low back or pelvic pain may or may not be related to prolapse.

The need for a standardized, reliable and clear staging method became more obvious in the last decades, with the increasing rate of scientific and professional interchanges, while the referral of patients to highly specialized centers is another issue supporting this need.

## A brief history of the classifications

Urogenital prolapse has traditionally been classified by the degree of anatomical deformity, depending on the site of the defect and the presumed pelvic viscera that are involved. The large number of different grading systems that have been used is reflective of the difficulty in designing an objective, reproducible system of grading prolapse. Intra– and interobserver variability is often important and may lead to confusion. This makes it difficult to compare successive examinations over time in the same woman or between different women.

**Table 1:** Traditional anatomical site prolapse classification

Urethrocele	Prolapse of the lower anterior vaginal wall involving the urethra only
Cystocele	Prolapse of the upper anterior vaginal wall involving the bladder. Generally there is also associated prolapse of the urethra and hence the term cystourethrocele is often used.
Uterovaginal Prolapse	This term is used to describe prolapse of the uterus, cervix and upper vagina
Enterocele	Prolapse of the upper posterior wall of the vagina usually containing loops of small bowel
Rectocele	Prolapse of the lower posterior wall of the vagina involving the rectum bulging forwards into the vagina

The other problem with this terminology is that it implies an unrealistic certainty as to the structures on the other side of the vaginal bulge. This is often a false assumption, particularly in women who have had previous prolapse surgery. The terms ‘anterior vaginal wall prolapse’, ‘posterior vaginal wall prolapse’ and ‘apical prolapse’ are therefore often preferred because of the uncertainty as to the anatomical structures on the other side of the vaginal bulge.

**Table 2:** Anatomical classification according to vaginal walls (enterocele can also be seen in the posterior compartiment)

Anterior Vaginal Wall (Anterior Compartment)	Cystocele	Urethrocele
	1. Central (Posterior)	Uncommon
	2. Lateral (Anterior)	
	3. Combined	
Apical Vaginal Wall (Middle Compartment)	Enterocele Uterine	Uterovaginal ; Vaginal vault
	1. Anterior	with cystocele, enterocele, rectocele; eversion (post-hysterectomy) with cystocele, enterocele, rectocele
	2. Posterior	
Posterior Vaginal Wall (Posterior Compartment)	Rectocele	
	1. Low	
	2. Midvaginal	
	3. High	
Perineal Body Defects		

The **Baden–Walker Halfway Scoring System** is the next system used, especially in clinical circumstances [5]. The assignment of a score to each of six specific midline sites encodes a large amount of information in a small amount of time and space. When descriptive notes and a pelvic organ prolapse map are added, a more complete description of the prolapse can be created. Although descriptive, some short–comings exist in the Baden–Walker system. For instance, a strategically placed 1cm–increase in prolapse results in an increase in the assigned stage. In addition, interobserver agreement is not perfect with the Baden–Walker system. 

**Figure 1:**
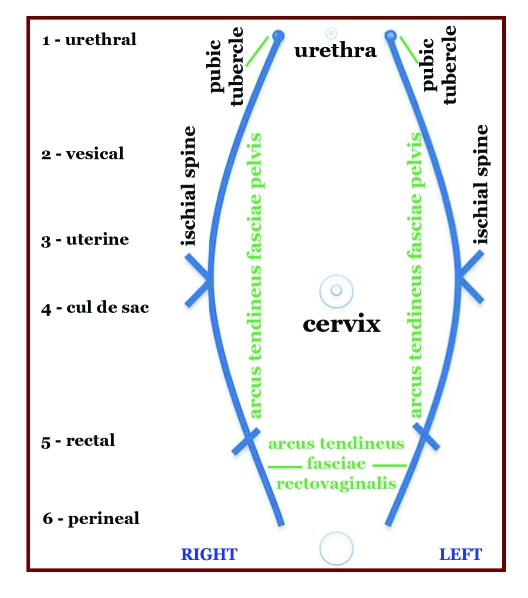
Pelvic organ prolapse map

By dividing the vagina through a coronal plane, tridimensional anatomy can be simplified to two dimensions. The key elements of pelvic support are described in Figure 1. The numbers represent the Baden–Walker vaginal support profile sites.

The extent of prolapse is recorded using a number (0 to 4) at each six sites in the vagina. Two sites are located on the anterior, superior and posterior walls of the vagina, respectively. Table 3 offers a list of anatomic sites and the associated symptoms. The six numbers are recorded as a measure of descent. For all sites except the perineum, the hymen is used as a fixed anatomic reference point. Zero indicates a normal anatomic position for a site, whereas 4 represents maximum prolapse. Between these extremes, the intervening numbers grade descent using a halfway system. The examination is performed with the patient straining so that maximum descent is attained. The perineum is graded using the familiar perineal laceration system used in obstetrics. The patient is asked to hold or strain to evaluate the amount of muscular and fascial support. Comments may include site of dominant prolapse, location of scars, palpable plications, and the type of efforts necessary to demonstrate maximum prolapse. Strength of the levator ani contraction may be recorded as 0 to 4.

For example, a pelvic support profile Baden–Walker is 12/44/32. This corresponds to a dominant complete proximal prolapse with enterocele, significant cystocele, and rectocele, and perineal attenuation to the level of the external sphincter. 2/4 levator ani strength is present. Although this type of notation encodes much information in a small space, no specific location of fascial defects is included [5].

**Figure 2:**
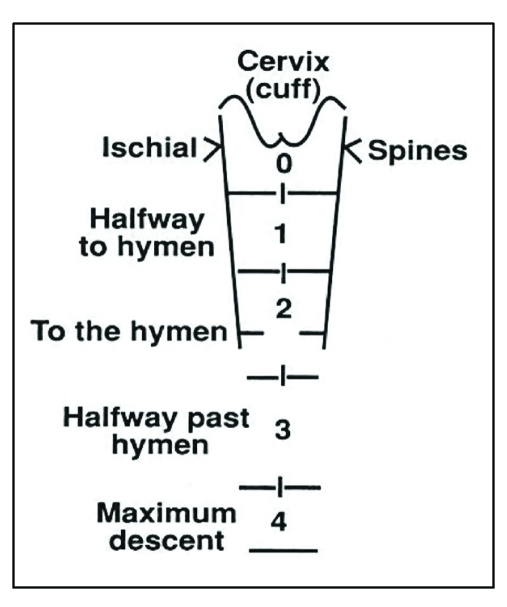
**Baden–Walker half way system **[6]. It consists of four grades: grade 0 – no prolapse, grade 1–halfway to hymen, grade 2 – to hymen, grade 3 – halfway past hymen, grade 4 –maximum descent.

In 1996, an article by Bump et al. [7] presents a standard system of terminology recently approved by the International Continence Society, the American Urogynecologic Society, and the Society of Gynecologic Surgeons for the description of female pelvic organ prolapse and pelvic floor dysfunction. An objective site–specific system for describing, quantifying, and staging pelvic support in women is included. It has been developed to enhance both clinical and academic communication regarding individual patients and populations of patients. Clinicians and researchers caring for women with pelvic organ prolapse and pelvic floor dysfunction are encouraged to learn and use the system.

In an effort to create an encoding tool useful to both the clinician and researcher, the Standardization Subcomitee of the International Continence Society created the Pelvic Organ Prolapse Quantification (POP–Q) system in 2002 [8,9,10,11].

The system relies on specific measurements of defined points in the midline of the vaginal wall. The fixed reference point used for measurement remains the hymeneal ring. In this system, small increases in prolapse are recorded as small increases in measurement. Because specific measurements at nine sites are recorded in a tic–tac–toe grid, interobserver agreement and reliability are also improved [12]. Researchers favor the use of POP–Q system for this reason. Unfortunately, the detail in making and recording nine measurements has been an impediment to more widespread clinical adoption of this system. However, it has been shown that the routine use of the POP–Q system decreases significantly the amount of time needed to collect the desired data [13]. Experienced examiners averaged 2.05 minutes per examination while new examiners averaged 3.73 minutes. There is also a high correlation between the POP–Q findings in left lateral and lithotomy position [14]. 

## How does the POPߝQ system works?

The hymen acts as the fixed point of reference throughout the POPQ system.

 There are six defined points for measurement in the POPQ system – Aa, Ba, C, D, Ap, Bp and three others landmarks: GH, TVL, PB. Each is measured in centimeters above or proximal to the hymen (negative number) or centimeters below or distal to the hymen (positive number) with the plane of the hymen being defined as zero (0). The hymen was selected as the reference point rather the introitus because it is more precisely identified [15].

**Figure 3:**
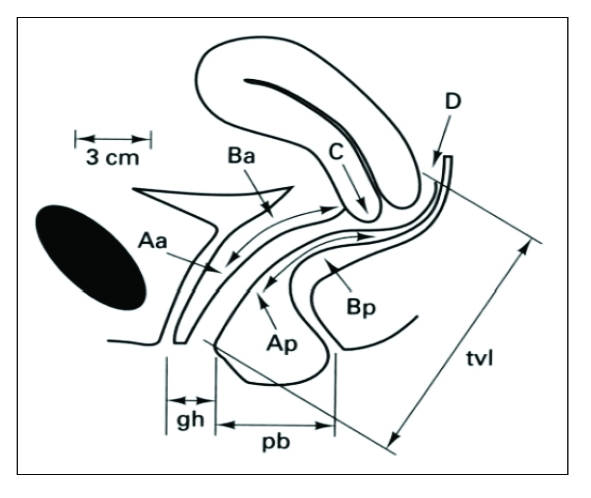
**Points and landmarks for POP–Q system examination.** Aa, point A anterior, Ap, point A posterior, Ba, point B anterior; Bp, point B posterior; C, cervix or vaginal cuff; D, posterior fornix (if cervix is present); gh, genital hiatus; pb, perineal body; tvl, total vaginal length

The terminology avoids assigning a specific label, such as cystocele or rectocele, to the prolapsing part of the vagina, acknowledging that the actual organ(s) above the prolapse frequently cannot be determined by physical examination. There are three reference points anteriorly (Aa, Ba, and C) and three posteriorly (Ap, Bp, and D). Points Aa and Ap are 3 cm proximal to or above the hymenal ring anteriorly and posteriorly, respectively. Points Ba and Bp are defined as the lowest points of the prolapse between Aa anteriorly or Ap posteriorly and the vaginal apex. Anteriorly, the apex is point C (cervix), and posteriorly is point D (pouch of Douglas). In women after hysterectomy, point C is the vaginal cuff and point D is omitted. Three other measurements are taken: the vaginal length at rest, the genital hiatus (gh) from the middle of the urethral meatus to the posterior hymenal ring, and the perineal body (pb) from the posterior aspect of the genital hiatus to the midanal opening.

**Figure 4:**
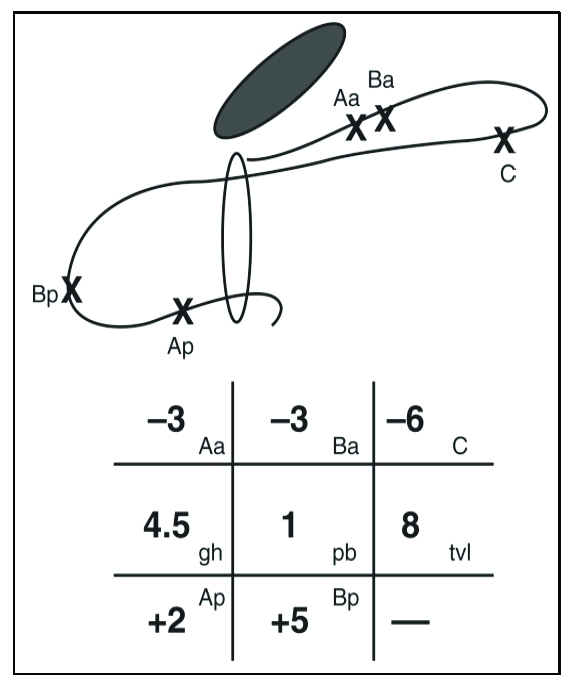
An example of measurements using the POP–Q system.

Grid and line diagrams of predominantly posterior support defect. Leading point of prolapse is upper posterior vaginal wall, point Bp (+5). Point Ap is 2 cm distal to hymen (+2) and vaginal cuff scar is 6 cm above hymen (–6). Cuff has undergone only 2 cm of descent because it would be at –8 (total vaginal length) if it were properly supported. This represents stage Ⅲ Bp prolapse.  *(From Bump RC, Mattiasson A, Bo K, et al: The standardization of terminology of female pelvic organ prolapse and pelvic floor dysfunction. Am J Obstet Gynecol 1996;175:10–17.)*

Once the measurements are taken, the patients are assigned to the corresponding stage:

**Table 3:** Stages of POP–Q system measurement

Stage 0	no prolapse is demonstrated
Stage 1	the most distal portion of the prolapse is more than 1 cm above the level of the hymen
Stage 2	the most distal portion of the prolapse is 1 cm or less proximal or distal to the hymenal plane
Stage 3	the most distal portion of the prolapse protrudes more than 1 cm below the hymen but protrudes no farther than 2 cm less than the total vaginal length (for example., not all of the vagina has prolapsed)
Stage 4	vaginal eversion is essentially complete

Excellent interobserver and intraobserver reliability has been shown [16]. It has been used for longitudinal follow–up of a population of women with prolapse [17] and extensively for outcome reporting after prolapse repair since 1996 [18]. However, there are some caveats. The system is more difficult to learn than the traditional staging and overall adoption by specialists is about 40% [19]. Patient position also affects reproducibility. The measurements are taken with the patient in the dorsal lithotomy position, and the degree of prolapse is assessed with patient straining. Prolapse may be more severe with the table raised at the head to a 45–degree angle [20]. The system also does not identify unilateral or asymmetrical defects. In 2006, this system was only used clinically by about 40% of members of ICS and AUGS. There has also been a developing of a POP–Q symplified system based on POP–Q with similar ordinal staging but with only four points measured instead of nine (Aa, Ba, C, D). Evaluation of the interobserver reproductibility and intersystems reliability (in comparisons with the standard POP–Q system) showed good correlation [21].

## Additional Testing

The initial evaluation of urinary incontinence in women includes history tacking, physical examination, urinalysis, and measurement of postvoid residual urine [22]. The basic evaluation may be satisfactory for proceeding with treatment, including surgery, for patients with straightforward stress incontinence associated with urethral hypermobility with normal postvoid residual volume [23]. However, the International Scientific Committee of the Third International Consultation on Urinary Incontinence advised that for women who desire interventional treatment, urodynamic testing is highly recommended [24].

Pelvic organ prolapse, as mentioned earlier, may be associated with LUTS and urodynamic findings of obstruction are demonstrable with flow rates and pressure/flow studies. The urodynamic level of outflow that defines obstruction in females is lower than in men [25]. Video–urodynamic and fluoroscopic studies, in addition to demonstrating incontinence and degree of hypermobility, may also allow characterization of the type of cystocele.

The role of routine cystoscopy in the evaluation of incontinence is controversial. Cystoscopy has also been reported to aid in the preoperative and intraoperative differentiation of the type of organ prolapse in patients with high–grade prolapse or multiple prolapsing organs [26]. It is done simply by identifying the light transmitted through the bladder wall. Intraoperative cystoscopy is also necessary to assess for bladder or urethral perforation or ureteric obstruction during various pelvic procedures.

Ultrasound imaging of the bladder and urethra can be done by the transabdominal, transperineal, translabial, transvaginal, or transrectal route. The advantage of ultrasound is the ability to do real–time scanning without radiation exposure, but the major disadvantages are the variability introduced by the examiner with small changes in the transducer position and the availability of only a limited number of pictures after the examination.

Two–dimensional translabial scanning is now a standard technique and has been reported for assessing position and mobility of the bladder neck and proximal urethra, stress incontinence, bladder wall thickness (with transvaginal scanning as well), levator ani activity (with perineal scanning), and prolapse quantification [27]. Multiple two–dimensional images can be combined, like slices of bread, to yield a three–dimensional image. Current transducers can acquire images by rapid oscillation of elements in a multitude of sectional planes within the transducer head. The images are integrated into a volume and displayed in various forms on a computer. Three–dimensional ultrasound has been used to image the urethra, levator ani complex, paravaginal supports, prolapse, and synthetic implant materials [28]. Ultrasound is not recommended in the primary evaluation of women with incontinence and prolapse and is an optional test for complex problems [29]

MRI may be helpful in patients with complex organ prolapse to supplement the physical examination. Its clinical utility in comparison with physical examination and in the decision for surgical management has yet to be demonstrated. MRI is not indicated in the evaluation of patients with incontinence or prolapse and is still considered an investigational tool [29]. Nevertheless, the dynamic MRI of the pelvic floor proved itself as an excellent tool for assessing functional disorders of the pelvic floor, including organ prolapse and incontinence. Recent studies suggest that dynamic MRI correlates very well with clinical examination in detection of the prolapse but may offer superior results when it comes to staging [30]. This investigation seems to be also useful in assessing the results of surgery for pelvic organ prolapse, even when the patient has no clinical symptoms.
